# Regulation of Osteogenic Markers at Late Stage of Osteoblast Differentiation in Silicon and Zinc Doped Porous TCP

**DOI:** 10.3390/jfb10040048

**Published:** 2019-11-05

**Authors:** Gary A. Fielding, Naboneeta Sarkar, Sahar Vahabzadeh, Susmita Bose

**Affiliations:** W. M. Keck Biomedical Materials Research Laboratory, School of Mechanical and Materials Engineering, Washington State University, Pullman, WA 99164-2920, USA; gfielding@wsu.edu (G.A.F.); naboneeta.sarkar@wsu.edu (N.S.);

**Keywords:** bone remodeling, calcium phosphate, osteoblast, silicon, zinc, differentiation markers

## Abstract

Calcium phosphates (CaPs) are one of the most widely used synthetic materials for bone grafting applications in the orthopedic industry. Recent trends in synthetic bone graft applications have shifted towards the incorporation of metal trace elements that extend the performance of CaPs to have osteoinductive properties. The objective of this study is to investigate the effects of silicon (Si) and zinc (Zn) dopants in highly porous tricalcium phosphate (TCP) scaffolds on late-stage osteoblast cell differentiation markers. In this study, an oil emulsion method is utilized to fabricate highly porous SiO_2_ doped β-TCP (Si-TCP) and ZnO doped β-TCP (Zn-TCP) scaffolds through the incorporation of 0.5 wt.% SiO_2_ and 0.25 wt.% ZnO, respectively, to the β-TCP scaffold. Reverse transcriptase quantitative polymerase chain reaction (RT-qPCR) is utilized to analyze the mRNA expression of osteoprotegerin (OPG), receptor activator of nuclear kappa beta ligand (RANKL), bone morphogenetic protein 2 (BMP2), and runt-related transcription factor 2 (Runx2) at the later stage of osteoblast differentiation, day 21 and day 28. Results show that the addition of Si and Zn to the β-TCP structure inhibited the β to α-TCP phase transformation and enhance the density without affecting the dissolution properties. Normal BMP-2 and Runx2 transcriptions are observed in both Si-TCP and Zn-TCP scaffolds at the initial time point, as demonstrated by RT-qPCR. Moreover, the addition of both Si and Zn positively regulate the osteoprotegerin: receptor activator of nuclear factor *k*-*β* ligand (OPG:RANKL) ratio at 21-days for Si-TCP and Zn-TCP scaffolds. These results demonstrate the effects of Si and Zn doped porous β-TCP scaffolds on the upregulation of osteoblast marker gene expression including OPG, RANKL, BMP-2, and Runx2, indicating the role of trace elements on the effective regulation of late-stage osteoblast cell differentiation markers.

## 1. Introduction

Calcium phosphate ceramics have been widely used in the orthopedic industry as synthetic bone graft substitutes, coating, and cement materials. They have an excellent biocompatibility and a compositional similarity to natural bone that make them ideal as bone graft substitutes [1,2]. Two of the major classes of calcium phosphates that are most often used and researched are hydroxyapatite (HA) and β-tricalcium phosphate (β-TCP). Due to the extremely low solubility of HA in physiological conditions, it is most often used in coating applications, where a long functional lifetime is necessary, or as a biphasic system as a mixture with β-TCP to increase the strength and longevity of grafting materials. β-TCP is about 30 orders of magnitude more soluble than HA, which makes it considered to be bioresorbable [3,4]. While HA may last in the human body for over 10 years, β-TCP has a functional lifetime of 12–16 months and is generally completely replaced by natural bone after 3 years [5,6]. β-TCP, thus, is an ideal material to fill small bony voids created during surgery or for spinal fusion and non-union fracture healing. β-TCP is considered by many to be an osteoconductive material. It provides support for new tissue formation and the migration of bone forming cells during the healing process. Recent trends in technology, however, demonstrate a paradigm shift from designing a simply functional material to a fully bioactive material. It has been the goal of many researchers and industry leaders to further develop β-TCP grafting materials to give them osteoinductive properties. In a study by Lindhorst et al., vascular endothelial growth factor (VEGF) was loaded onto β-TCP scaffolds and was found to increase neovascularization in vivo, a vital characteristic of the bone healing process [7]. Several studies have outlined the efficacy of recombinant human bone morphogenic protein 2 (rhBMP-2) loaded TCP scaffolds, demonstrating enhanced osteogenic differentiation of human adipose-derived stem *cells in vitro* in as soon as 15 min and significantly improved new bone formation in vivo [8,9]. Other growth factors often investigated, including transforming growth factors (TGFs) and fibroblast growth factors (FGFs), have shown similar positive results [10,11]. While much of these results have been extremely positive, growth factors have come under severe scrutiny of the public and the Food and Drug Administration (FDA) due to several serious possible side effects experienced in clinical off label use [12,13].

An alternative approach of incorporating osteoinductive capabilities to β-TCP has been through the use of trace elements that are vital to bone development and health, such as silicon (Si) and zinc (Zn). Si has long been understood to play an important role in bone and connective tissue biology [14]. In a study by Seaborn and Nielsen, Si-deprived rats were shown to have significantly decreased collagen formation in bone and wound healing [15]. Another study noted a positive correlation with dietary Si uptake and bone mineral density in the lumbar vertebrae of men and premenopausal women [16]. In addition to the osteogenic effects of Si, it has also demonstrated angiogenic capabilities. Human dermal fibroblasts indicated upregulated VEGF production in response to calcium silicates, which had a downstream effect on the nitric oxide synthase in human endothelial cells [17]. Zn is another trace element of importance in bone biology. Zn^2+^ is one of the ions that are released during the bone remodeling process and plays a crucial role in the stability and activity of alkaline phosphatase (ALP) [18]. Excess Zn release during the remodeling process is believed to play a role in the regulation of osteoclast activity as well as stimulation of osteoblast activity [19,20].

We have previously reported that the use of these trace elements can alter the physicochemical properties of β-TCP such as compressive strength, strength degradation, grain size, and density. Furthermore, the presence of these dopants has been shown to enhance in vitro and in vivo biological responses of calcium phosphates [21,22,23,24,25,26]. While the beneficial effects of Si and Zn have been demonstrated in various in vitro and in vivo studies, little is known as to their primary mechanism of action when compared to commonly used growth factors. In this work, we have studied the effects of Si and Zn dopants on the regulation of osteogenic markers such as OPG, RANKL, RUNX2, and BMP2 at the late stage of osteoblastic differentiation. In this study, highly porous TCP scaffolds are doped with Si or Zn in order to investigate the expression of osteoblast gene markers at late-stage differentiation, where confluent monolayer of osteoblast cells is observed on doped scaffolds as well as the positive regulation of osteogenic markers expressions are noted at day 21 and 28. 

## 2. Materials and Methods 

### 2.1. Sample Preparation and Characterization

β-tricalcium phosphate powder (β-TCP) was synthesized via a solid-state method. Briefly, 2 moles of dicalcium phosphate (CaHPO_4_) were mixed with 1 mole of calcium carbonate (CaCO_3_) for 2 h in a 5:1 milling media to powder weight ratio. The mixture was calcined at 1050 °C for 24 h, followed by cooling to room temperature. High purity silicon dioxide (SiO_2_) (99%+ purity) and zinc oxide (ZnO) (99.9%+ purity) were purchased from Fisher Scientific (Fair Lawn, NJ, USA). Powders were prepared by mixing 20 g of β-TCP powder and appropriate amounts of dopants (0.5 wt.% SiO_2_ and 0.25 wt.% ZnO) in 250 mL polypropylene Nalgene bottles containing 30 mL of anhydrous ethanol and 100 g zirconia milling media with 5mm diameter. Dopant concentrations were chosen based on previous optimization research. The mixtures were then milled for 6 h at 70 rpm to minimize the formation of agglomerates and increase homogeneity. After milling, powder was dried in an oven at 70 °C for 72 h. Porous samples were prepared using an oil emulsion method as previously described. Briefly, 20g of β-TCP powder (pure or doped) were mixed with 20 g paraffin oil purchased from Sigma Aldrich (St. Louis, MO, USA) and 13.4 mL of an emulsifier solution of 0.14g/L Kolliphor EL purchased from Sigma Aldrich (St. Louis, MO, USA) in 0.2 M Na_2_HPO_4_. The mixture was stirred at 2000 rpm for 45 sec to yield a stable emulsion slurry. The slurry was then pipetted into disk (11.43 mm diameter and 2.35 mm thickness) or cylinder-shaped molds (6 mm diameter and 12 mm height), followed by drying at 70 °C. Samples were then removed from the molds and sintered at 1250 °C for 2 h in a muffle furnace.

The surface morphology of the sintered scaffolds before and after immersion in Phosphate Buffered Saline (PBS) with pH of 7.4 was characterized using a Field Emission Scanning Electron Microscope (FESEM) (FEI Inc., OR, USA) before and after 28 days of incubation in PBS. Phase analysis of sintered pure and doped TCP samples was carried out by Siemens (Aubrey, TX, USA) D500 Krystalloflex X-ray diffractometer (XRD) using Cu Kα radiation at 35 kV and 30 mA at room temperature, over the 2θ range between 20° and 40° at a step size of 0.1° and a count time of 1 sec per step. Relative bulk and apparent densities were measured using the Archimedes’ method. Samples were weighed initially dry and then submerged in boiling water for 3 min to remove any excess air that may be trapped in the porous structure. The samples were then transferred from the boiling water to room temperature water, where the weight was recorded again (n = 3). Compressive strengths of pure and doped TCP scaffolds were determined using a screw-driven universal testing machine (AG-IS, Shimadzu, Japan) with a constant crosshead speed of 0.33 mm/min. Compressive strength was calculated using the maximum recorded load and the sample dimensions. Compressive strength was tested on at least five samples for each composition.

### 2.2. Cell Culture and Cellular Morphology

All samples were sterilized by autoclaving at 121 °C for 60 min before cell culture. An aseptic condition was maintained during the entire cell work. For this study, established human pre-osteoblast cell line hFOB 1.19 was purchased from ATCC, Manassas, VA, USA. The base medium for this cell line was a 1:1 mixture of Ham’s F12 Medium and Dulbecco’s Modified Eagle’s Medium (DMEM/F12, Sigma, St. Louis, MO, USA), with 2.5 mM L-glutamine (without phenol red). The medium was supplemented with 10% fetal bovine serum (HyClone, Logan, UT, USA) and 0.3 mg/ml G418 (Sigma, St. Louis, MO, USA). The hFOB cell is cultured in T75 culture flasks until they reach 70-80% confluency. The sterilized samples were kept in 24-well plates and hFOB cells were seeded onto the samples at a density of 2 × 10^5^ cells/sample. 1 ml of base medium was added in each well containing samples and the medium was changed every 2–3 days for the duration of the experiment. Cultures were maintained at 34 °C under an atmosphere of 5% CO_2_ as recommended by ATCC for this particular cell line. 

Samples for testing were removed from culture after 21 and 28 days of incubation. All samples for SEM observation were fixed with 2% paraformaldehyde/2% glutaraldehyde in 0.1 M phosphate buffer overnight at 4 °C. Post-fixation was performed with 2% osmium tetroxide (OsO_4_) for 2 h at room temperature. The fixed samples were then dehydrated in an ethanol series (30%, 50%, 70%, 95%, and 100% three times), followed by a hexamethyldisilane (HMDS) drying procedure. After gold coating, the samples were observed under FESEM for cell morphologies. 

### 2.3. RNA Extraction and Real-Time RT-PCR

RNA was extracted from samples using Aurum Total RNA Mini Kit spin columns from BioRad (Hercules, CA, USA) and the manufacturer’s recommended procedure is followed. Three biological replicates were used in this study with each having three technical replicates. First strand cDNA was synthesized using an iScript Advanced cDNA Synthesis Kit for RT-qPCR (Biorad, Hercules, CA, USA) in 20 μL reactions, according to the manufacturer’s recommended procedure. RT-qPCR was performed under standard enzyme and cycling conditions on a CFX Connect Real-Time PCR Detection System (Biorad, Hercules, CA, USA). Primer sets were pre-validated by PrimePCR SYBR Green Assays from Biorad (Hercules, CA, USA) for BMP-2 (assay ID: qHsaCID0015400), runt-related transcription factor 2 (Runx2; assay ID: qHsaCID0006726), osteoprotegerin (OPG; assay ID: qMmuCID0027158) and receptor activator of nuclear factor kappa-B ligand (RANKL; assay ID: qHsaCID0015585). Two housekeeping genes β-actin (assay ID: qHsaCED0036269) and ribosomal protein, large, P0 (RPLP0; assay ID: qHsaCED0036271) were also used in this study. Data analysis was performed using the BioRad CFX Manager Software 3.0 (Hercules, CA, USA). Expression levels of the gene of interest were normalized to the housekeeping genes and data reported is the normalized expression given by 2^−ΔΔ𝐶𝑡^. 

### 2.4. Statistical Analysis

All experimental data are representative of at least three biological and three technical replicates. Quantitative data for gene expression is reported as mean ± standard deviation. Statistical analysis was performed using two-way ANOVA and Bonferroni post-hoc analysis was carried out in a GraphPad Prism 8 software (Hercules, CA, USA). *p* < 0.05 was considered statistically significant. 

## 3. Results

### 3.1. Physical and Mechanical Characterization

**Figure 1:**
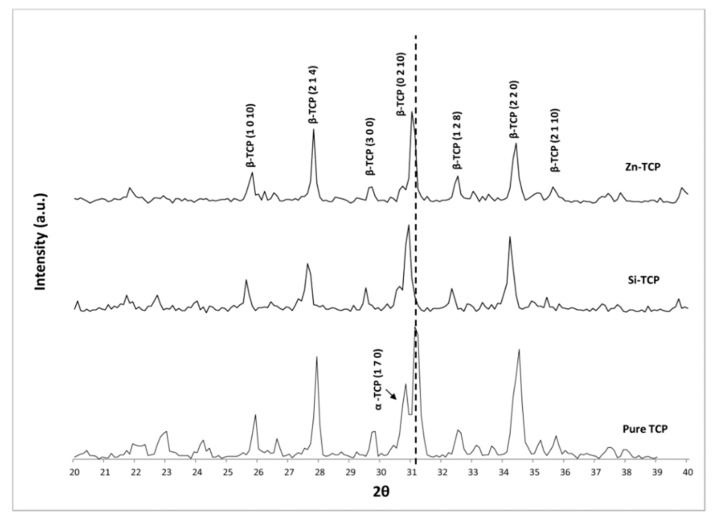
XRD patterns of doped and pure scaffolds sintered at 1250 °C showing characteristics peaks of primary phase, β-TCP in doped TCP. JCPDS # 09-0169 (β-TCP) and 09-0348 (α-TCP).

Figure 1 shows the XRD patterns, which is performed to determine the CaP phases of the sintered scaffolds and confirm that in both samples, β-TCP is the primary phase present after sintering at 1250 °C. α-TCP phase is found to be present in the pure samples, but drastically reduced in the samples containing dopants. Zn-TCP has the least amount of α-TCP, while pure TCP has the most. The characteristic peaks of β-TCP and α-TCP match well with JCPDS# 09-0169 (β-TCP) and 09-0348 (α-TCP), but show about a 1.57 degree peak shift in Zn-TCP samples and a 1.79 degree peak shift in the Si-TCP samples. 

**Figure 2:**
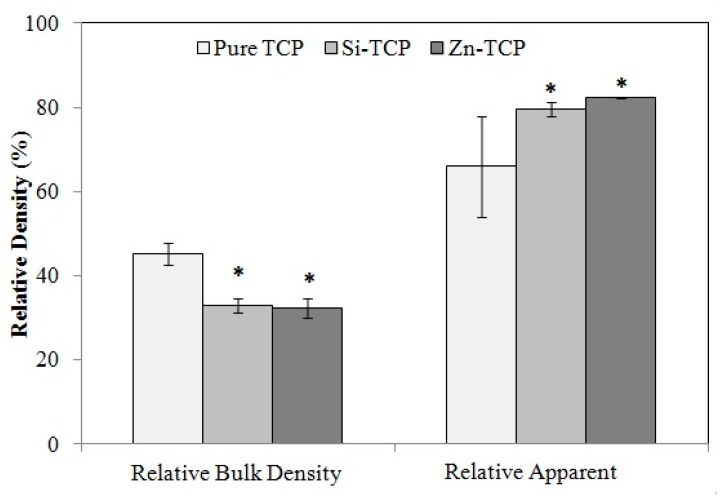
Relative bulk and apparent densities of sintered porous samples showing significantly higher apparent density in doped samples. Statistical analysis shows that the differences are significant (* *p* < 0.05, where n = 3).

Figure 2 shows relative bulk and apparent densities of samples, respectively. Pure TCP has the highest relative bulk density of 45.14% ± 0.03%, whereas the bulk density of Si-TCP and Zn-TCP are 32.81% ± 0.02% and 32.4% ± 0.02%, respectively. Relative apparent density measurement shows that Zn-TCP and Si-TCP are 82.36% ± 0.02% and 79.64% ± 0.02% dense. Pure TCP has the lowest relative density of 66.05% ± 0.12%. 

**Figure 3:**
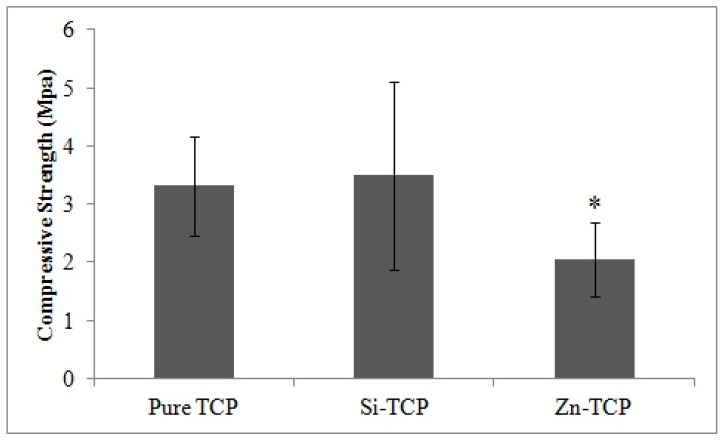
Compressive strength of sintered porous samples showing. Statistical analysis shows that the differences are significant (* *p* < 0.05, where n = 5).

Compressive strength of sintered scaffolds is shown in Figure 3. Pure TCP and Si-TCP has similar compressive strength of 3.31 ± 0.85 MPa and 3.50 ± 1.61 MPa, respectively. Zn-TCP has compressive strength of 2.06 ± 0.64 MPa, significantly lower than the two other compositions.

**Figure 4:**
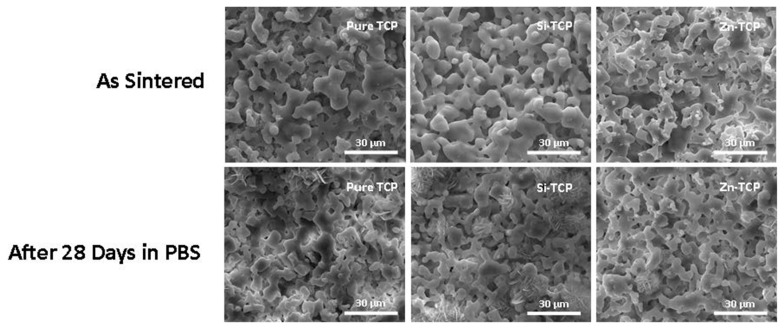
Surface morphology of sintered scaffolds of pure TCP composition, Si-TCP and Zn-TCP compositions after sintering and after 28 days in PBS showing enhanced apatite formation in presence of dopants, compared to the pure TCP scaffold.

FESEM micrographs of samples before and after 28 days of immersion in PBS are shown in Figure 4, demonstrating the highly porous nature of the scaffolds. All samples show evidence of liquid phase sintering, characterized by the flowing particle structures. After 28 days immersion in PBS, all samples have shown considerable surface degradation and significant plate-like apatite formation, but the microstructure remain largely unchanged. Si-TCP samples show the most apatite formation followed by Zn-TCP samples, while the pure TCP samples has the least amount of apatite. 

**Figure 5:**
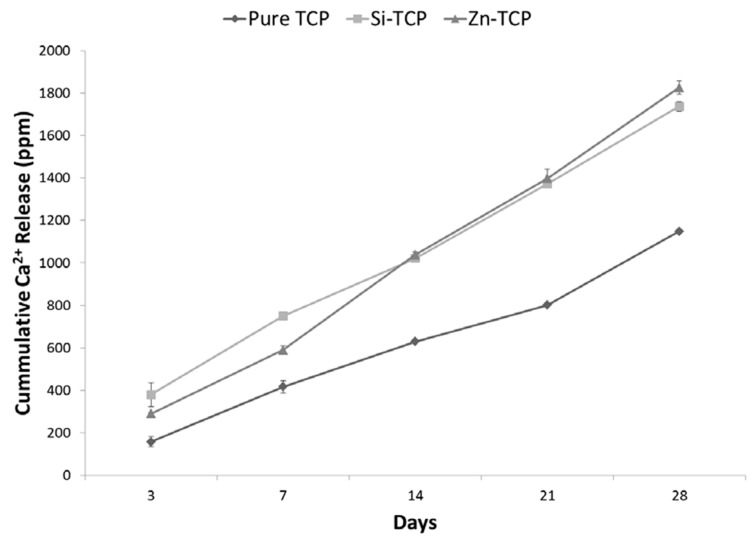
Atomic Absorption Spectroscopy (AAS) results for Ca^2+^ ion concentration in PBS collected over 28 days showing higher degradation rate of Zn-TCP and Si-TCP scaffolds, compared to the pure TCP scaffold.

Ca^2+^ ion release results in Figure 5 indicate both of the doped compositions has higher degradation rates than the pure scaffolds. In total, Si-TCP samples release 1.74 mg of Ca^2+^ over 28 days. Zn-TCP samples has an average 1.83 mg Ca^2+^ over 28 days, while the pure samples has only 1.15 mg Ca^2+^ release. All samples has a steady state of release over the testing period.

**Figure 6:**
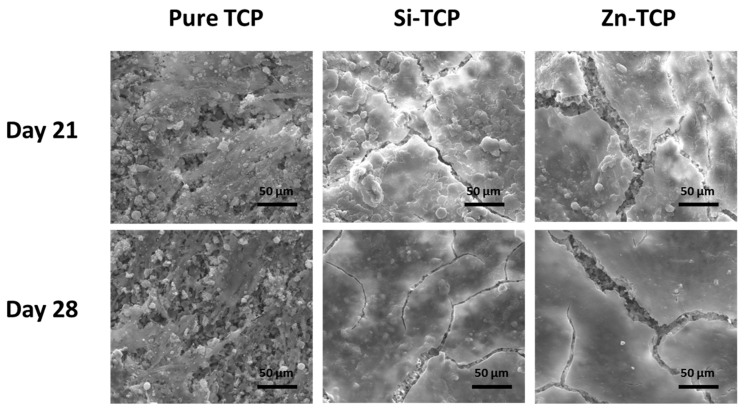
FESEM micrographs depicting hFOB cell morphology after 21 and 28 days in culture showing layer-like osteoblast cellular morphology on doped samples indicating enhanced osteoblast cell proliferation in presence of dopants, compared to pure TCP.

Figure 6 shows the morphology of hFOB cells on pure and doped samples after 21 and 28 days of culture. While single cells are difficult to identify on doped samples, the pure samples shows presence of some, indicating a higher proliferation rate in the Si-TCP and Zn-TCP scaffolds. 

### 3.2. Osteoblast Cell Gene Expression Analysis

**Figure 7:**
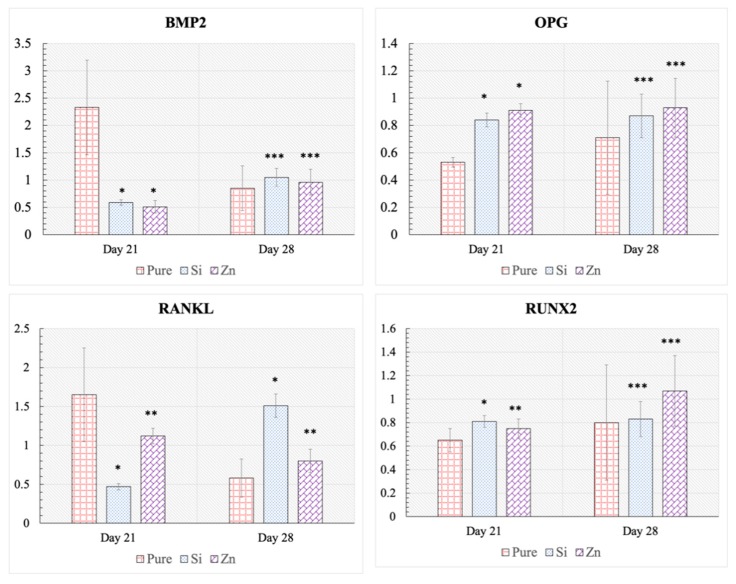
mRNA expression for BMP2, OPG, RANKL, and RUNX2 analyzed by RT-qPCR at day 21 and 28 for pure and doped TCP scaffolds showing significant upregulation of OPG, and downregulation of RANKL in presence of dopants at day 21, compared to the pure TCP. (* *p* < 0.001, ** *p* < 0.5, *** not statistically significant, where n = 3).

**Figure 8:**
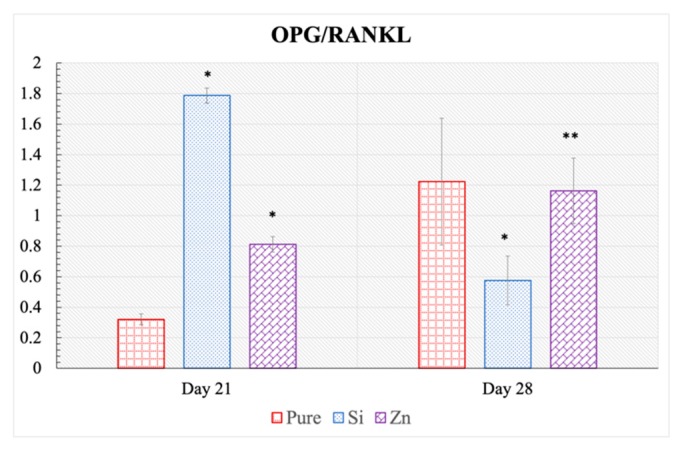
The OPG/RANKL ratio at day 21 and 28 showing positive regulation of OPG:RANKL ratio by doped samples at the initial time point of 21 days.(* *p* < 0.001, ** *p* < 0.5, where n = 3).

Osteoblast gene expression analysis results after 21 and 28 days of culture are presented in Figure 7. At day 21, Si-TCP and Zn-TCP have expressed significantly less BMP-2 than the pure samples. OPG expression is upregulated significantly in the doped samples, while a significant downregulation of RANKL is noted in Si-TCP and Zn-TCP samples when compared to the pure samples. Runx2 expression is similar at day 21 for all samples, with elevated expression measured in the Si-TCP samples. However, at day 28, similar BMP-2 and OPG expressions are seen in all samples, compared to the control. RANKL expression is elevated for Si-TCP samples, while Zn-TCP has exhibited a statistically higher expression to the pure composition. Runx2 expression in Si-TCP and Zn-TCP are similar to pure TCP at day 28 with no statistically significant difference noted. Additionally, the OPG:RANKL ratio are calculated for control, Si-TCP and Zn-TCP samples at day 21 and 28, as shown in Figure 8. At day 21, the OPG:RANKL ratio is significantly upregulated in doped samples, compared to the control TCP. Finally, at day 28, the OPG:RANKL ratio has shown a decrease in trend for Si-TCP, however on Zn-TCP, a gradual but statistically lower increase has been observed, compared to the control.

## 4. Discussion

The use of Si and its result as a stabilizing agent in calcium phosphates (CaPs) has been well documented [27,28]. XRD results shown in Figure 1 confirm that α-TCP phase formation is significantly reduced in samples containing Si and Zn. It is believed that Si^4+^ replaces P^5+^ in the TCP lattice with charge compensation created by either the presence of excess Ca^2+^ or O^2−^ vacancies [29]. Zn^2+^, however, is most likely to replace Ca^2+^ in Ca(5) position in the lattice structure where it is believed that the Ca(5)O_6_ polyhedron may be strained due to over-bonding and would favor the incorporation of smaller cations such as zinc [30]. Both dopants seem to stabilize the crystal structure during sintering at high temperatures (>1150 °C) where α-TCP is known to form. A slight peak shift is detectable in both doped samples, verifying that substitution defects are likely taking place. 

Density analysis is in agreement with the XRD results. α-TCP phase formation is known to coincide with a stage of rapid grain growth and an overall decrease in the densification [31,32]. Higher α-TCP phase amount in pure sample results in a less densified scaffold with 66% relative apparent density, while doped samples show relative densities of over 79%. Microstructural analysis by SEM (Figure 4) verifies that all the samples have greater than 20% open pores with most of the porosity being micropores. While all samples have evidence of liquid phase formation, Si-TCP samples exhibit a significantly increased amount. Si (r = 111 pm) has a slightly larger atomic radius than that of phosphorous (r = 98 pm), which causes bond elongation and strain in the crystal structure that could result in a decrease in liquidus temperature and increase in liquid phase sintering [33]. 

The mechanical strength of the pure TCP is 3.31 ± 0.85 MPa. Addition of Zn decreases the compressive strength to 2.06 ± 0.64 MPa, while the Si dopant does not affect it significantly. Previous studies have reported a decrease in initial compressive strength of ZnO doped compact TCP samples [34], which can be attributed to the slight differences in the emulsion formation during processing and resultant variances in pore size and distribution rather than an inherent physicochemical property associated with Zn doping in this system. The driving factor in the compressive strength in this study is far more weighted by a large amount of porosity than the physical effects of the dopants.

After 28 days of immersion in PBS, all samples show signs of surface degradation, but the microstructure does not change significantly (Figure 4). The only calcium phosphate product that is stable in solution at a pH greater than 4.2 is hydroxyapatite (HA) [35]. The dissolution-precipitation process of β-TCP has been described as a diffusion limited process, which will decrease as an interfacial apatite layer is formed on the surface of the sample [36]. While all samples show apatite formation, Si-TCP and Zn-TCP have significantly more surface apatite formation than the pure samples, indicating a faster rate towards the dissolution-precipitation equilibrium. 

The osteoblast cells go through three stages in their lifecycle: proliferation, maturation, and differentiation. The early stage is focused on the population and recruitment of cells which leads to the maturation stage. During the maturation stage, cells lay the foundation for new bone called the extracellular matrix (ECM). Once the ECM is created, cells will begin terminal differentiation or undergo apoptosis [37]. SEM images in Figure 6 exhibit that doped samples form a layer-like osteoblast cellular morphology at day 21, compared to the control. There is no significant difference between cellular morphology at day 28 compared to day 21. 

Osteoblasts play a dual role in the bone remodeling process. Aside from depositing the ECM, they can regulate osteoclastogenesis via OPG and RANKL production. After successfully depositing the new matrix, osteoblasts need to send a signal to monocytes to begin the formation of merged and multinucleated osteoclasts. This signal has been identified as RANKL and is considered the primary cause of osteoclastogenesis. OPG is a decoy receptor for RANKL that is produced by osteoblasts. Generally, when RANKL is upregulated it is associated with a downregulation of OPG, so as to favor osteoclastogenesis and the ratio is considered to be a major determinant of bone mass [38].

In the present study, RT-qPCR results show significantly lower BMP-2 expression in the doped samples, indicating the osteoblast differentiation process has been already initiated in Si-TCP and Zn-TCP samples before day 21. BMP2 is reported to be known as an early phase osteoblast differentiation marker, which attributes to the downregulation of this gene in doped samples at day 21. Upregulation of BMP2 in control samples at day 21 enables us to confirm late initiation of osteoblast differentiation in pure TCP samples. Similarly, it has been demonstrated that Runx2 expression needs to be upregulated for the differentiation of MSCs into osteoblasts but downregulated for terminal differentiation of osteoblasts into bone lining cells and osteocytes [19]. In this regard, day 21 results from this study show that Runx2 transcriptional activity is relatively less, yet statistically higher compared to the pure TCP control group. Additionally, the significant upregulation of OPG and marked downregulation of RANKL in doped samples at day 21 support their pivotal role in bone regeneration. The pure TCP samples at day 21, however, indicate the opposite response where high RANKL expression and low OPG expression would result in the induction of bone remodeling by osteoclast cells. 

RT-qPCR expression of osteoblast markers at day 28 suggests that the cells on the pure TCP samples are undergoing the differentiation process, which is indicated by lower BMP-2 levels, moderate Runx2 expression, and a high OPG: RANKL ratio. The osteoblasts cultured on both the Si-TCP and Zn-TCP seem to already have gone through differentiation with no significant difference in BMP2, Runx2, and OPG expression between the control and doped samples. 

Interestingly, we see that the OPG: RANKL ratio remains significantly high in both the Si-TCP and Zn-TCP at day 21, indicating an affinity for prolonged bone growth processes and delayed osteoclastogenesis. The markedly higher OPG/RANKL ratio for Si doped samples at day 21 indicates a better bone remodeling cycle leading to accelerated healing. However, day 28 results show a decrease in RANKL expression for pure TCP as well as an increase in the same for Si-TCP and Zn-TCP, resulting in a significantly smaller OPG:RANKL ratio for doped samples. These results indicate that the osteoblasts on doped samples were mostly differentiated. 

**Figure 9:**
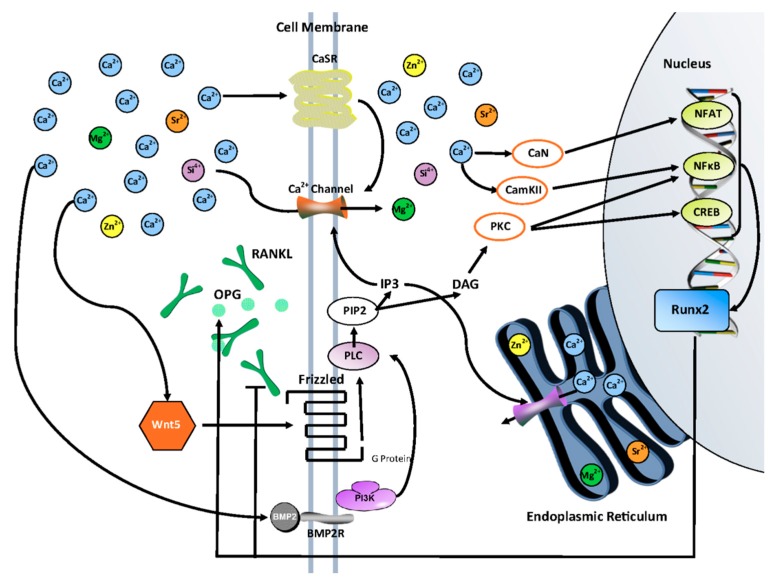
Activation of the Wnt/Fz ligand-receptor leads to the production of the second messengers IP3 and DAG from membrane-bound PIP2 via the action of membrane-bound enzyme PLC. IP3 causes a release of Ca^2+^ from the ER and extracellular Ca^2+^ influx through transmembrane Ca^2+^ channels; CaN and CamKII are activated which in turn activate NFAT and NFkB. DAG is also activated by increased intracellular Ca^2+^, which activates PKC. PKC activates NFkB and CREB. NFAT, NFkB, and CREB translocate to the nucleus and transcribe downstream regulatory genes such as Runx2. Elevated Runx2 activity increases OPG production while simultaneously decreasing RANKL production. BMP2, upregulated by extracellular Ca^2+^, can affect this pathway by binding to BMP2 receptors which activate PI3k. PI3k activates the gamma subunit of PLC increasing its conversion of IP3 and DAG [23,41].

It has been verified that OPG and RANKL are both regulated by the Wnt signaling pathway, which is one of the main pathways in osteogenesis, as well as by intracellular calcium signaling [39,40]. In a previous study, we found that the use of dopants in compact TCP samples effectively modulated post-translational Runx2 activity, having elevated levels during the proliferation stage and decreased levels during the maturation stage of the osteoblast lifecycle [18,20]. OPG has been shown to be regulated by the Wnt pathway, intracellular Ca^2+^ signaling and Runx2, RANKL by Runx2 and intracellular Ca^2+^ signaling, BMP-2 by PI-3K and PKC and Runx2 by intracellular Ca^2+^ signaling, they all have a common relationship with intracellular calcium (Figure 9). Dopants may act as competitive agonists with Ca^2+^ in many of these pathways and disrupt normal function due to size and/or charge differences, causing altered but a beneficial expression of important targets [23]. 

Nevertheless, future studies need to be performed to investigate the Wnt and NFkβ expressions to confirm the potential association between the gene expression and the mechanistic pathway. Further advancements can also be achieved by analyzing the expression of osteoblast differentiation markers at early time points. Additionally, future research involving in vivo studies are necessary to evaluate the effect of these dopants on the osteoblast phenotype. 

## 5. Conclusions

CaP materials are one of the most widely utilized and extensively studied materials for use as synthetics in orthopedic medicine. Incorporation of Si and Zn into highly porous calcium phosphate scaffolds resulted in denser scaffold with relative apparent density of 82.4% and 79.6% for Zn-TCP and Si-TCP, respectively. Moreover, the addition of dopants mitigated the formation of soluble α-TCP phase at high sintering temperatures while not affecting the dissolution properties of the scaffolds. SEM and RT-PCR results demonstrate that the addition of both Si and Zn dopants stimulated osteoblast cell differentiation while positively regulating the osteoblast marker genes. For instance, Si-TCP and Zn-TCP demonstrated a statistically significant upregulation in OPG expression and a downregulation in RANKL expression at the initial time point of 21 days. Moreover, the presence of Si and Zn maintained an increased OPG:RANKL ratio at the initial time points, indicating their key importance in the enhancement of osteoblast cell differentiation as well as positive regulation of bone metabolism.

## References

[1] Hoover S., Tarafder S., Bandyopadhyay A., Bose S. (2017). Silver doped resorbable tricalcium phosphate scaffolds for bone graft applications. Mater. Sci. Eng. C.

[2] Nandi S.K., Fielding G., Banerjee D., Bandyopadhyay A., Bose S. (2018). 3D-printed β-TCP bone tissue engineering scaffolds: Effects of chemistry on in vivo biological properties in a rabbit tibia model. J. Mater. Res..

[3] Aulakh T.S., Jayasekera N., Kuiper J.-H., Richardson J.B. (2009). Long-term clinical outcomes following the use of synthetic hydroxyapatite and bone graft in impaction in revision hip arthroplasty. Biomaterials.

[4] Li S., de Wijn J.R., Li J., Layrolle P., de Groot K. (2003). Macroporous biphasic calcium phosphate scaffold with high permeability/porosity ratio. Tissue Eng..

[5] Iezzi G., Malchiodi L., Quaranta A., Ghensi P., Piattelli A. (2013). Peri-implant bone response around a human hydroxyapatite-coated implant retrieved after a 10-year loading period: A case report. Int. J. Oral Maxillofac. Implants.

[6] Tanaka T., Kumagae Y., Saito M., Chazono M., Komaki H., Kikuchi T., Kitasato S., Marumo K. (2008). Bone formation and resorption in patients after implantation of beta-tricalcium phosphate blocks with 60% and 75% porosity in opening-wedge high tibial osteotomy. J. Biomed. Mater. Res. B Appl. Biomater..

[7] Lindhorst D., Tavassol F., von See C., Schumann P., Laschke M.W., Harder Y., Bormann K.-H., Essig H., Kokemüller H., Kampmann A. (2010). Effects of VEGF loading on scaffold-confined vascularization. J. Biomed. Mater. Res. A.

[8] Kim J.-W., Jung I.-H., Jeong I.-H., Lee K.-I., Jung U.-W., Kim C.-S., Choi S.-H., Cho K.-S., Yun J.-H. (2012). Volumetric bone regenerative efficacy of biphasic calcium phosphate-collagen composite block loaded with rhBMP-2 in vertical bone augmentation model of a rabbit calvarium. J. Biomed. Mater. Res. A.

[9] Overman J.R., Farré-Guasch E., Helder M.N., ten Bruggenkate C.M., Schulten E.A.J.M., Klein-Nulend J. (2013). Short (15 minutes) bone morphogenetic protein-2 treatment stimulates osteogenic differentiation of human adipose stem cells seeded on calcium phosphate scaffolds in vitro. Tissue Eng. Part A.

[10] Komaki H., Tanaka T., Chazono M., Kikuchi T. (2006). Repair of segmental bone defects in rabbit tibiae using a complex of beta-tricalcium phosphate, type I collagen, and fibroblast growth factor-2. Biomaterials.

[11] Ripamonti U., Teare J., Ferretti C. (2012). A Macroporous Bioreactor Super Activated by the Recombinant Human Transforming Growth Factor-β(3). Front. Physiol..

[12] Carragee E.J., Hurwitz E.L., Weiner B.K. (2011). A critical review of recombinant human bone morphogenetic protein-2 trials in spinal surgery: Emerging safety concerns and lessons learned. Spine J. Off. J. N. Am. Spine Soc..

[13] James A.W., LaChaud G., Shen J., Asatrian G., Nguyen V., Zhang X., Ting K., Sooet C. (2016). A review of the clinical side effects of bone morphogenetic protein-2. Tissue Eng. Part B Rev..

[14] Vu A.A., Robertson S.F., Ke D., Bandyopadhyay A., Bose S. (2019). Mechanical and biological properties of ZnO, SiO_2_, and Ag_2_O doped plasma sprayed hydroxyapatite coating for orthopaedic and dental applications. Acta Biomater..

[15] Seaborn C.D., Nielsen F.H. (2002). Silicon deprivation decreases collagen formation in wounds and bone, and ornithine transaminase enzyme activity in liver. Biol. Trace Elem. Res..

[16] Jugdaohsingh R., Tucker K.L., Qiao N., Cupples L.A., Kiel D.P., Powell J.J. (2004). Dietary silicon intake is positively associated with bone mineral density in men and premenopausal women of the Framingham Offspring cohort. J. Bone Min. Res. Off. J. Am. Soc. Bone Min. Res..

[17] Li H., Chang J. (2013). Bioactive silicate materials stimulate angiogenesis in fibroblast and endothelial cell co-culture system through paracrine effect. Acta Biomater..

[18] Yamaguchi M. (2010). Role of nutritional zinc in the prevention of osteoporosis. Mol. Cell. Biochem..

[19] Vahabzadeh S., Bandyopadhyay A., Bose S., Mandal R., Nandi S.K. (2015). IGF-loaded silicon and zinc doped brushite cement: Physico-mechanical characterization and in vivo osteogenesis evaluation. Integr. Biol..

[20] Ke D., Tarafder S., Vahabzadeh S., Bose S. (2019). Effects of MgO, ZnO, SrO, and SiO_2_ in tricalcium phosphate scaffolds on in vitro gene expression and in vivo osteogenesis. Mater. Sci. Eng. C.

[21] Bandyopadhyay A., Petersen J., Fielding G., Banerjee S., Bose S. (2012). ZnO, SiO_2_, and SrO doping in resorbable tricalcium phosphates: Influence on strength degradation, mechanical properties, and in vitro bone-cell material interactions. J. Biomed. Mater. Res. B Appl. Biomater..

[22] Fielding G.A., Bandyopadhyay A., Bose S. (2012). Effects of SiO2 and ZnO doping on mechanical and biological properties of 3D printed TCP scaffolds. Dent. Mater..

[23] Fielding G.A., Smoot W., Bose S. (2014). Effects of SiO_2_, SrO, MgO, and ZnO dopants in tricalcium phosphates on osteoblastic Runx2 expression. J. Biomed. Mater. Res. A.

[24] Fielding G., Bose S. (2013). SiO_2_ and ZnO dopants in three-dimensionally printed tricalcium phosphate bone tissue engineering scaffolds enhance osteogenesis and angiogenesis in vivo. Acta Biomater..

[25] Bose S., Fielding G., Tarafder S., Bandyopadhyay A. (2013). Understanding of dopant-induced osteogenesis and angiogenesis in calcium phosphate ceramics. Trends Biotechnol..

[26] Roy M., Fielding G., Bandyopadhyay A., Bose S. (2013). Effects of Zinc and Strontium Substitution in Tricalcium Phosphate on Osteoclast Differentiation and Resorption. Biomater. Sci..

[27] Ning C.Q., Greish Y., El-Ghannam A. (2004). Crystallization behavior of silica-calcium phosphate biocomposites: XRD and FTIR studies. J. Mater. Sci. Mater. Med..

[28] Fuh L.J., Huang Y.J., Chen W.C., Lin D.J. (2017). Preparation of micro-porous bioceramic containing silicon-substituted hydroxyapatite and beta-tricalcium phosphate. Mater. Sci. Eng. C.

[29] Bose S., Banerjee D., Robertson S., Vahabzadeh S. (2018). Enhanced in vivo bone and blood vessel formation by iron oxide and silica doped 3D printed tricalcium phosphate scaffolds. Ann. Biomed. Eng..

[30] Wei X., Akinc M. (2007). Crystal Structure Analysis of Si- and Zn-Codoped Tricalcium Phosphate by Neutron Powder Diffraction. J. Am. Ceram. Soc..

[31] Kanazawa T., Umegaki T., Yamashita K., Monma H., Hiramatsu T. (1991). Effects of additives on sintering and some properties of calcium phosphates with various Ca/P ratios. J. Mater. Sci..

[32] Ryu H.-S., Youn H.-J., Hong K.S., Chang B.-S., Lee C.-K., Chung S.-S. (2002). An improvement in sintering property of beta-tricalcium phosphate by addition of calcium pyrophosphate. Biomaterials.

[33] Pichavant M. (1987). Effects of B and H_2_O on liquidus phase relations in the haplogranite system at l kbar. Am. Miner..

[34] Bandyopadhyay A., Bernard S., Xue W., Bose S. (2006). Calcium Phosphate-Based Resorbable Ceramics: Influence of MgO, ZnO, and SiO_2_ Dopants. J. Am. Ceram. Soc..

[35] Driessens F., Verbeeck R. (1990). Biominerals.

[36] Ishikawa K. (2010). Bone substitute fabrication based on dissolution-precipitation reactions. Materials.

[37] Bradley E.W., Westendorf J.J., van Wijnen A.J., Dudakovic A. (2018). Osteoblasts: Function, Development, and Regulation. Primer on the Metabolic Bone Diseases and Disorders of Mineral Metabolism.

[38] Boyce B.F., Xing L. (2007). Biology of RANK, RANKL, and osteoprotegerin. Arthritis Res. Ther..

[39] Boyce B.F., Xing L., Chen D. (2005). Osteoprotegerin, the bone protector, is a surprising target for beta-catenin signaling. Cell Metab..

[40] Zhang L., Liu W., Zhao J., Ma X., Shen L., Zhang Y., Jin F., Jin Y. (2016). Mechanical stress regulates osteogenic differentiation and RANKL/OPG ratio in periodontal ligament stem cells by the Wnt/β-catenin pathway. Biochim. Biophys. Acta Gen. Subj..

[41] MacLeod R.J., Hayes M., Pacheco I. (2007). Wnt5a secretion stimulated by the extracellular calcium-sensing receptor inhibits defective Wnt signaling in colon cancer cells. Am. J. Physiol. Gastrointest. Liver Physiol..

